# Synthesis of γ-hydroxy-α-(arylmethyl)carboxylic acids from lactones: pathway to a structural motif derived from lactic acid and amino acid analogs?

**DOI:** 10.1186/s13104-019-4232-1

**Published:** 2019-04-02

**Authors:** Nicole Panzier, Fabian Uhrner, Felix Lederle, Jan C. Namyslo, Eike G. Hübner

**Affiliations:** Institute of Organic Chemistry, Clausthal University of Technology, Leibnizstr. 6, 38678 Clausthal-Zellerfeld, Germany

**Keywords:** Lactones, α-Angelica lactone, γ-Hydroxy-α-(arylmethyl)carboxylic acids, Lactic acid, Amino acid analogs, Poly(lactic acid)

## Abstract

**Objective:**

A synthetic pathway to γ-hydroxy-α-(arylmethyl)carboxylic acids starting from α-angelica lactone and γ-butyrolactone was investigated. These γ-hydroxycarboxylic acids resemble structural motifs of lactic acid and amino acids. The possibility of cocondensation with lactic acid towards functionalized poly(lactic acid)s was analyzed.

**Results:**

The functional γ-hydroxycarboxylates sodium 4-hydroxy-2-((*N*-methylpyrazol-4-yl)methyl)pentanoate and sodium 4-hydroxy-2-(phenylmethyl)butanoate (2-benzyl-4-hydroxybutanoate) were synthesized in good yields as a proof of concept for the proposed reaction pathway. Additionally, sodium (*E*)-2-((*N*-methylpyrazol-4-yl)methylene)-4-oxopentanoate presenting an interesting structural motif was isolated. All products have been fully characterized by mass spectrometry, IR spectroscopy and 2D nuclear magnetic resonance (NMR) techniques. In contrast to the carboxylate anions, the corresponding carboxylic acids obtained after acidification were found to be unstable. The instability was analyzed by NMR experiments. With the help of diffusion ordered NMR spectroscopy, the cocondensation with lactic acid was elucidated. The reaction products were characterized as oligomers of pure lactic acid, while intramolecular condensation of the γ-hydroxycarboxylic acids prevents cocondensation with lactide.

**Electronic supplementary material:**

The online version of this article (10.1186/s13104-019-4232-1) contains supplementary material, which is available to authorized users.

## Introduction

**Fig. 1:**
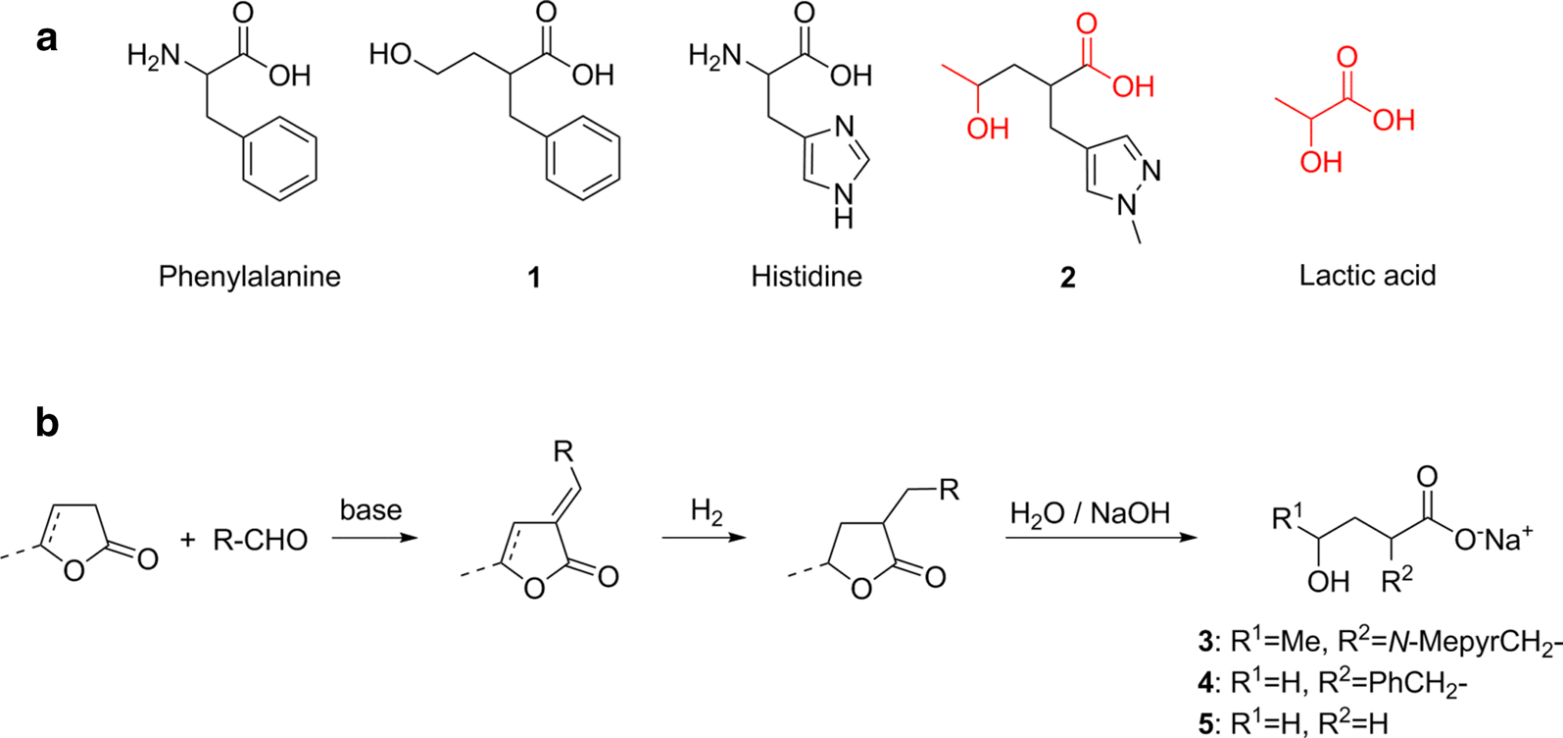
Pathway to γ-hydroxy-α-(aryl/alkylmethyl)carboxylates. **a** Structural motif of 4-hydroxy-2-(phenylmethyl)butanoic acid **(1)** (2-benzyl-4-hydroxybutanoic acid) in comparison to phenylalanine and 4-hydroxy-2-((*N*-methylpyrazol-4-yl)methyl)pentanoic acid (**2**) in comparison to histidine and lactic acid. **b** General synthetic pathway starting from α-angelica lactone or y-butyrolactone

We report on a convenient pathway to γ-hydroxy-α-(arylmethyl)carboxylates. Starting from lactones such as α-angelica lactone, treatment with carboxaldehydes such as benzaldehyde is known to lead to α-arylidenefuran-2-ones (Fig. 1b) [1]. Recently, we investigated the application of *N*-heterocyclic carboxaldehydes [2]. y-Butyrolactone or y-valerolactone can be used as starting materials, too and yield various derivatives [3–5]. Ring-opening of the lactone by saponification leads to a γ-hydroxy-α-(aryl/alkylmethyl)carboxylate (Fig. 1b). The structural motif of γ-hydroxypentanoic acid resembles the structure of lactic acid elongated by a C_2_-spacer unit (Fig. 1a). The alkyl/arylmethyl substitution in α-position resembles the structural motif of various amino acids (Fig. 1a). For example, R^1^ = H and R^2^ = benzyl (Fig. 1b) corresponds to the 2-hydroxyethyl analog (**1**) of phenylalanine (Fig. 1a). Amino acid analogs are of interest for various reasons and derivatives with an hydroxy instead of the amino moiety are of potential use, e.g. for unnatural peptides [6, 7]. Our motivation for the synthesis of 4-hydroxy-2-((*N*-methylpyrazol-4-yl)methyl)pentanoic acid (**2**) was an investigation of its cocondensation with lactic acid. The condensation of lactic acid, in comparison to the ring-opening polymerization of lactide, is an important technique especially for the large scale synthesis of poly(lactic acid) (PLA) [8]. Functionalized PLAs are of interest in view of enhanced material properties and biological or medical activity [9–11]. We are interested in the introduction of biocompatible (biodegradable) *N*-donor moieties in the PLA chain to coordinate various metal ions [12] and to achieve a thermal stabilization via metal ion crosslinking [13].

## Main text

### Experimental section

The synthesis of the precursors (*E*)-5-methyl-3-((*N*-methylpyrazol-4-yl)methylene)furan-2-one (**7**), 5-methyl-3-((*N*-methylpyrazol-4-yl)methyl)dihydrofuran-2-one (**9**), and (*E*)-3-benzylidendihydrofuran-2-one (**6**) has been performed according to our previous publication [2]. 3-Benzyldihydrofuran-2-one (**8**) has been prepared according to literature [14].

Details concerning equipment, purification of starting materials and procedure for polycondensation are given in Additional file 1.

### General procedure for saponification

Equimolar amounts of aqueous NaOH (*c* = 1–2 wt%) and lactone are slowly heated in a small round bottom flask under continuous stirring to reflux conditions. After 3 h, the solution is cooled down, extracted 3× with chloroform to remove impurities and the water phase is reduced in vacuo. The resulting salt is dried in high vacuo.

### Synthesis of sodium 4-hydroxy-2-((*N*-methylpyrazol-4-yl)methyl)pentanoate (**3**)

According to the general procedure, NaOH (0.680 g, 16.99 mmol) and 5-methyl-3-((*N*-methylpyrazol-4-yl)methyl)dihydrofuran-2-one (**9**) (3.300 g, 16.99 mmol) yield sodium 4-hydroxy-2-((*N*-methylpyrazol-4-yl)methyl)pentanoate (**3**) as slightly yellow solid. Yield: 3.900 g (98%).

The full characterization including all ^1^H and ^13^C NMR, IR and mass spectral data is given in Additional file 1.

### Synthesis of sodium 4-hydroxy-2-(phenylmethyl)butanoate (**4**) (2-benzyl-4-hydroxybutanoate)

According to the general procedure, NaOH (0.023 g, 0.567 mmol) and 3-benzyldihydrofuran-2-one (**8**) (0.100 g, 0.567 mmol) yield sodium 4-hydroxy-2-(phenylmethyl)butanoate (**4**) as white solid. Yield: 0.083 g (68%).

The full characterization including all ^1^H and ^13^C NMR, IR and mass spectral data is given in Additional file 1.

### Synthesis of sodium 4-hydroxybutanoate (**5**)

According to the general procedure, NaOH (0.233 g, 5.816 mmol) and γ-butyrolactone (dihydrofuran-2-one) (0.501 g, 5.816 mmol) yield sodium 4-hydroxybutanoate (**5**) as white solid. Yield: 0.682 g (93%).

The full characterization including all ^1^H and ^13^C NMR, IR and mass spectral data is given in Additional file 1.

### Synthesis of sodium (*E*)-2-((*N*-methylpyrazol-4-yl)methylene)-4-oxopentanoate (**10**)

According to the general procedure, NaOH (0.105 g, 2.63 mmol) and (*E*)-5-methyl-3-((*N*-methylpyrazol-4-yl)methylene)furan-2-one (**7**) (0.500 g, 2.63 mmol) yield sodium (*E*)-2-((*N*-methylpyrazol-4-yl)methylene)-4-oxopentanoate (**10**) as red solid. Yield: 0.269 g (45%).

The full characterization including all ^1^H and ^13^C NMR, IR and mass spectral data is given in Additional file 1.

## Results and discussion

**Fig. 2:**
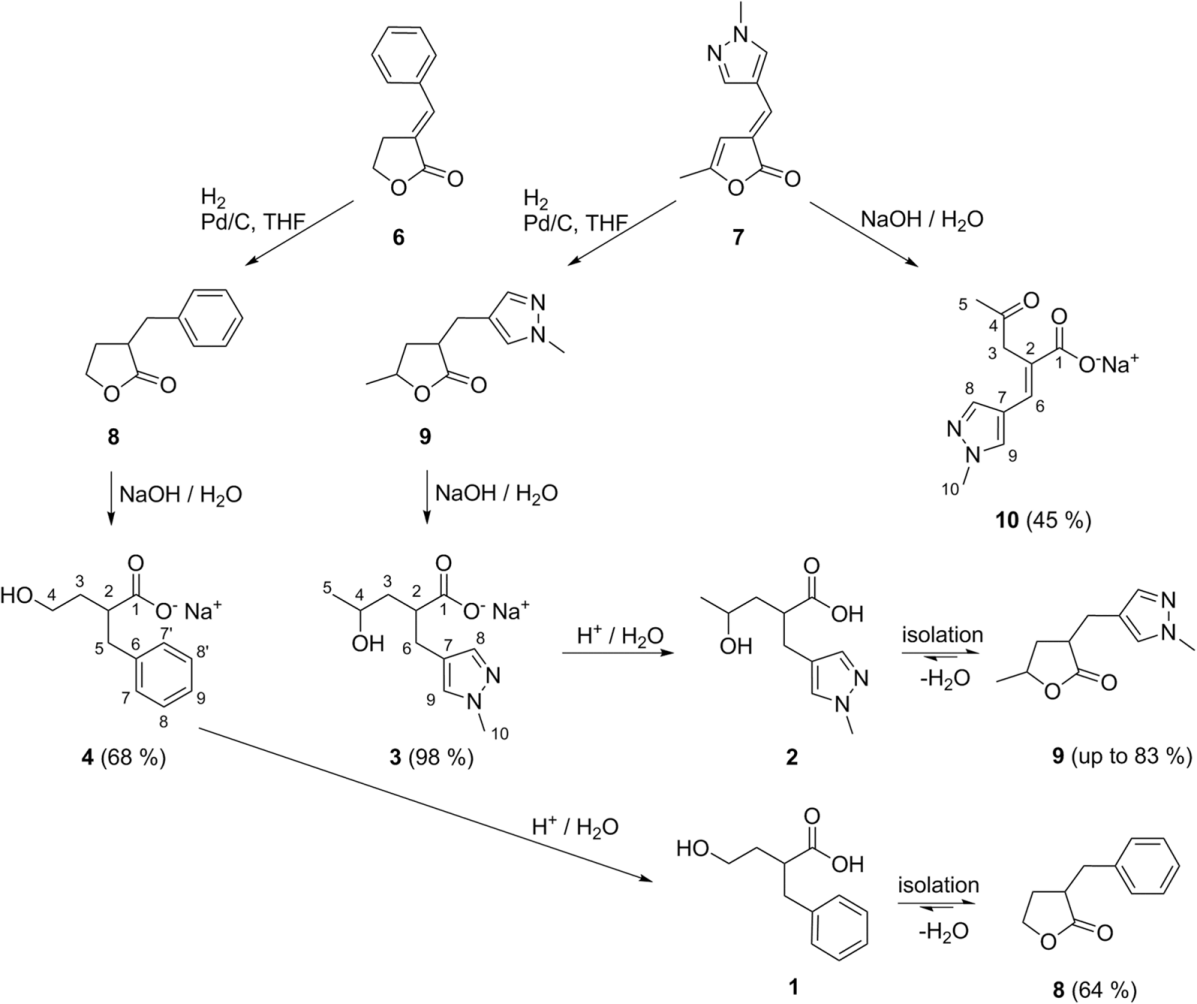
Synthesis of 4-hydroxy-2-(arylmethyl)carboxylates starting from (*E*)-3-benzylidendihydrofuran-2-one (**6**) and (*E*)-5-methyl-3-((*N*-methylpyrazol-4-yl)methylene)furan-2-one (**7**), hydrogenation to 3-benzyldihydrofuran-2-one (**8**) and 5-methyl-3-((*N*-methylpyrazol-4-yl)methyl)dihydrofuran-2-one (**9**) and finally saponification to sodium 4-hydroxy-2-(phenylmethyl)butanoate (**4**) and sodium 4-hydroxy-2-((*N*-methylpyrazol-4-yl)methyl)pentanoate (**3**)

α-Angelica lactone has been identified as one of the key components from biomass to valuable chemical products [15]. Recently, we presented the synthesis of the *N*-donor functionalized (*E*)-5-methyl-3-((*N*-methylpyrazol-4-yl)methylene)furan-2-one (**7**) from α-angelica lactone [2]. Here, we investigated ring-opening of the lactone ring towards functionalized γ-hydroxycarboxylates. Treatment of **7** with NaOH/H_2_O yields sodium 4-hydroxy-2-((*N*-methylpyrazol-4-yl)methylene)pent-3-enoate, which immediately isomerizes to its tautomer sodium (*E*)-2-((*N*-methylpyrazol-4-yl)methylene)-4-oxopentanoate (**10**) in 45% yield (not optimized) (Fig. 2). The formation of **10** is clearly indicated by two signals at 207.3 (CO) and 171.4 (CO_2_^−^) in the ^13^C nuclear magnetic resonance (NMR) spectrum as well as the carbonyl vibration at 1652 cm^−1^ in the infrared (IR) spectrum. **10** is in possession of a versatile and stiff structural motif with a 1,4-diketone unit, a monoanionic carboxylate donor and an oppositely orientated neutral *N*-donor, making it an interesting candidate for metal organic frameworks.

As the tautomerism towards the keto-form prohibits the use of **10** as bifunctional comonomer, **7** was hydrogenated to **9** according to procedures given in literature [2]. Subsequent ring-opening of the lactone yields the sodium salt (**3**) of the corresponding γ-hydroxy-α-(arylmethyl)pentanoic acid in excellent yields of 98%. Formation of the anion **3** is proven by the signal at m/z = 211.1 in the electrospray ionization (ESI) mass spectra (anion mode) as well as the broad signal at 6.73 ppm in the ^1^H NMR spectrum for the hydroxy group. Owed to the diastereotopic protons in position 3 and 6 (see Fig. 2 for labeling) and resulting inequivalent protons in the ^1^H NMR spectra, the NMR spectrum of **3** is rather crowded. Nevertheless, all protons could be identified and all couplings were resolved unambiguously with the help of 2D-NMR techniques. To check the general applicability of the pathway, the sequence was repeated starting from benzaldehyde and γ-butyrolactone and subsequent hydrogenation of the resulting (*E*)-3-benzylidendihydrofuran-2-one (**6**) to 3-benzyldihydrofuran-2-one (**8**). Final saponification of the lactone ring leads to the sodium salt (**4**) of the corresponding γ-hydroxy-α-(arylmethyl)butanoic acid in a yield of 68%. Again, the formation of **4** is clearly indicated by the signal at m/z = 193.1 in the mass spectra (anion mode) as well as the full set of NMR data.

### Acidification towards the free acids

The protonation of **3** and **4** to the free acids **1** and **2** was not straightforward. Protonation of **3** with several methods only allowed to isolate the corresponding lactone **9** in moderate to good yields (HCl and extraction with chloroform at various pH values: 20%; ion exchange column: 64%; HCl, drying and soxhlet extraction with THF: 83%). Even working with exact equimolar amounts of the sodium salt **5** and freshly titrated HCl led to a mixture of lactone **9**, the free acid **1** and sodium chloride from which only lactone **9** could be isolated. The same observation is valid for the carboxylate **4**, from which solely lactone **8** was isolated in 64% yield after acidification.

In literature, the instability of γ-hydroxycarboxylic acids has been reported early [16]. Recent examples describe the recovery of the lactone by extraction after protonation of the γ-hydroxycarboxylate [17]. On the other hand, the isolation of the free γ-hydroxycarboxylic acid after acidification of the potassium salt and subsequent extraction is reported, too [18]. Commonly, the γ-hydroxycarboxylate is protonated in situ [19] or the crude mixture after acidification of the γ-hydroxycarboxylate is directly used for further transformations [20, 21]. An interesting aspect is the potential stabilization of the γ-hydroxycarboxylic acid by basic moieties of the molecular structure and formation of a zwitterionic species [22]. Although **1** is in possession of a nitrogen donor, the formation of a tautomer with a pyrazolium cation and carboxylate anion is rather unlikely. According to the p*K*_S_ values, the protonated *N*-methylpyrazolium cation (p*K*_S_: 3.55) is more acidic than γ-hydroxybutyric acid (p*K*_S_: 4.71) or β-phenylpropionic acid (p*K*_S_: 4.64) as comparable carboxylic acids [23]. Consequently, a significant fraction of **1** should be present as neutral species which matches with the observed formation of the lactone **9**.

**Fig. 3:**
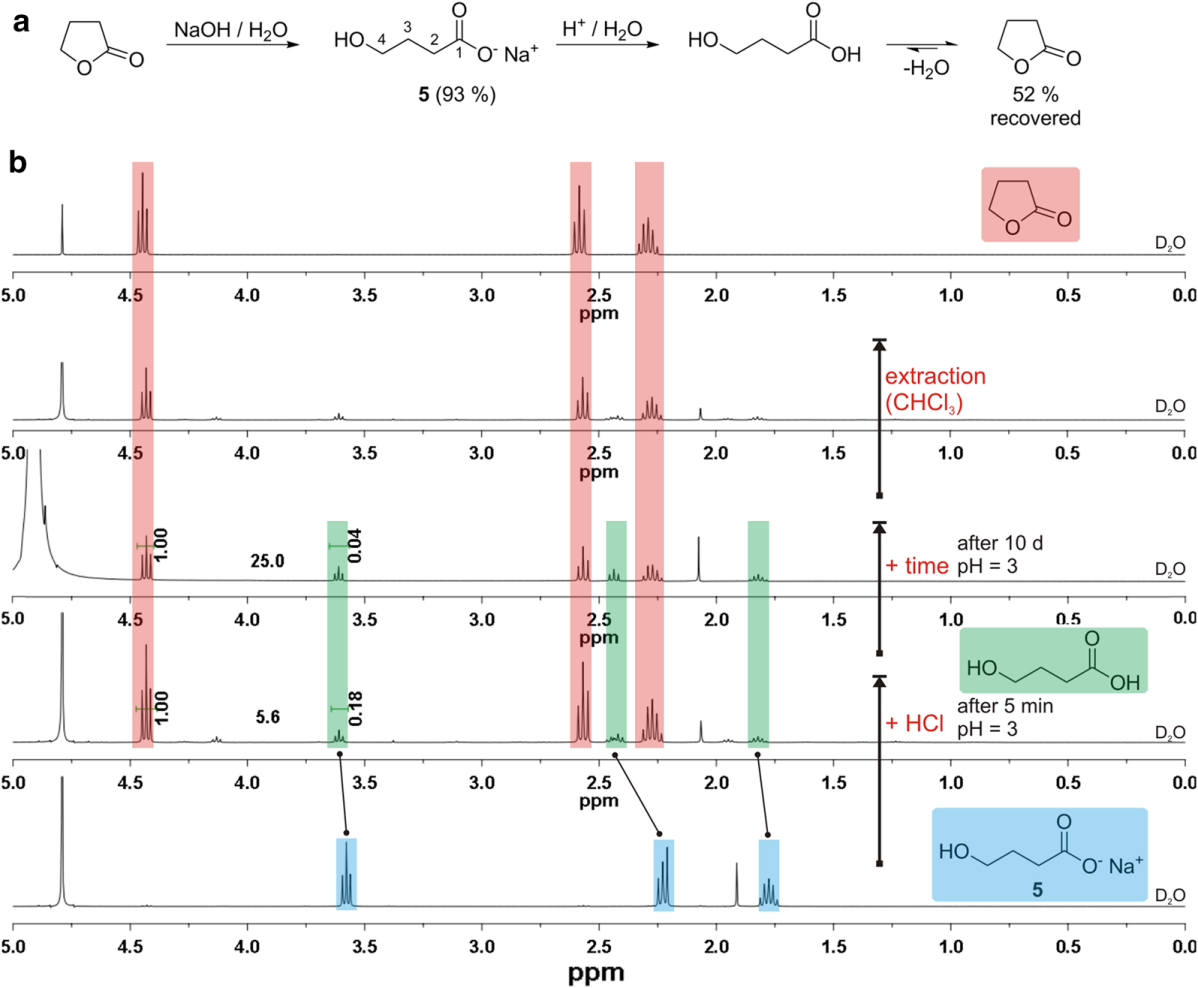
Formation of γ-butyrolactone from sodium 4-hydroxybutanoate (**5**). **a** Synthesis of **5** from γ-butyrolactone and recovery of the lactone after acidification. **b** ^1^H NMR spectra of **5** (bottom), after addition of HCl (pH = 3) directly after the addition and after 10 days, ^1^H NMR of the reaction product obtained after extraction of the water phase with chloroform and pure γ-butyrolactone (top). All spectra measured in D_2_O

Since results in literature report the immediate formation of the lactone as well as the isolation of the γ-hydroxycarboxylic acid after protonation, here we performed an NMR experiment based on γ-butyrolactone as the basic structural motif (Fig. 3). The ^1^H NMR spectra of sodium 4-hydroxybutanoate (**5**) in D_2_O revealed solely the γ-hydroxycarboxylate (Fig. 3b). Subsequently, HCl was added up to pH = 3 to ensure complete protonation of the carboxylate. Immediately after addition, and within the water phase, intramolecular condensation towards γ-butyrolactone (85%) took place. Obviously, the reaction is not substantially kinetically hindered since a second measurement after 10 days did not strongly change the composition. The extraction of this mixture with chloroform led to nearly pure γ-butyrolactone (Fig. 3b). The long-time interconversion of γ-butyrolactone and γ-hydroxybutyric acid at ambient conditions in aqueous media, with a focus on beverages at varying pH values, has been investigated more detailed in literature [24].

### Polycondensation

Finally, the cocondensation of **2** with lactic acid was investigated. Since acidification of **3** and isolation of the product leads to the lactone **9** as described above, sodium 4-hydroxy-2-((*N*-methylpyrazol-4-yl)methyl)pentanoate (**3**) was treated with equimolar amounts of HCl and the resulting raw mixture was used as comonomer. Of course, the strongly acidic medium provided by lactic acid (p*K*_S_: 3.86 [23]) can be assumed to promote the intramolecular ring closure even further. On the other hand, acid-catalyzed ester hydrolysis of the lactone ring can be expected [24], especially at the elevated temperatures of the polycondensation reaction. First, polycondensation conditions given in literature [25] were adapted and optimized in terms of catalyst concentration and temperature profile towards a small batch size of 5 g. The protocol for all polycondensation reactions is given in Additional file 1. With 1 mol% titanium *n*-butoxide as catalyst and at 180 °C, pure poly(lactic acid) with a molecular weight of *M*_n_ = 10,600 g/mol (*M*_w_ = 15,500 g/mol) (rel. polystyrene calibration) was achieved. Subsequently, the condensation was repeated after addition of 5 and 10 mol% of the acidified mixture of **3**. Size exclusion chromatography (SEC) revealed a lower molecular weight (*M*_n_ = 2600 g/mol) which increased to *M*_n_ = 4100 g/mol at longer reaction times (see Additional file 1: Table S1 for the results of all cocondensation reactions). To further analyze the reaction product, diffusion ordered NMR spectroscopy (DOSY) measurements of the crude mixture after polycondensation were performed. As visualized in Additional file 1: Figure S3, several oligomers of pure lactic acid, identified by the ^1^H NMR signals around 1.55 ppm and 5.15 ppm, were formed. The DOSY data revealed a highest molecular weight fraction with a diffusion coefficient around 2.2 × 10^−10^ m^2^/s and a main fraction between *D* = 2 × 10^−10^ m^2^/s and 4 × 10^−10^ m^2^/s, roughly matching with the results from SEC (*D* = 3 × 10^−10^ m^2^/s is recalculated to approx. 3300 g/mol). The signals assigned to **2** or the corresponding lactone **9**, identified by the pyrazol protons around 7.2 and 7.3 ppm, belong to a very fast diffusing species (8 × 10^−10^ m^2^/s) and consequently a monomeric molecule.

Overall, the results allow concluding that the hydroxycarboxylic acid **2** is not incorporated into the PLA chain. This can be readily explained by the preferred intramolecular condensation towards the corresponding lactone **9**. The slight decrease of the molecular weights obtained in presence of **2**/**9** can be explained by *N*-coordination of the nitrogen donor towards the metal catalyst.

## Limitations

- Starting from α-angelica lactone and γ-butyrolactone an efficient pathway to γ-hydroxy-α-(arylmethyl)carboxylates which resemble the structural motif of lactic acid and amino acid analogs was investigated. As proof of concept, sodium 4-hydroxy-2-((*N*-methylpyrazol-4-yl)methyl)pentanoate (**3**) and sodium 4-hydroxy-2-(phenylmethyl)butanoate (**4**) were synthesized in three steps.
- The free γ-hydroxy-α-(arylmethyl)carboxylic acids obtained after protonation of the carboxylates were found to be unstable due to immediate intramolecular condensation towards the corresponding lactones.
- The application as comonomer for the polycondensation with lactic acid towards functional poly(lactic acid)s was found to be not possible.

## Additional file

**Additional file 1.** Supporting information contains details concerning equipment, measurement parameters, suppliers of chemical substances, purification/drying of starting materials, procedure for polycondensation reactions, molecular weight of all polycondensation products, all analytical data for compounds **3**, **4**, **5** and **10**, DOSY NMR spectra of **9** and the polycondensation product of lactic acid in presence of **3** after equimolar acidification as well as raw ^1^H and ^13^C NMR spectra of **3**, **4**, **5** and **10.**

## Abbreviations

DMSO: dimethyl sulfoxide
DOSY: diffusion ordered spectroscopy
ESI: electrospray ionization
IR: infrared
NMR: nuclear magnetic resonance
PLA: poly(lactic acid)
SEC: size exclusion chromatography
THF: tetrahydrofurane

## References

[1] Egorova AY, Reshetov PV, Morozova NA, Sedavkina VA (1997). 3-Arylidene derivatives of 3H-furan-2-ones. Synthesis and reaction with maleic anhydride. Chem Heterocycl Compd..

[2] Uhrner F, Lederle F, Namyslo JC, Gjikaj M, Schmidt A, Hübner EG (2017). Reaction of *N*-heterocyclic carbaldehydes with furanones—an investigation of reactivity and regioselectivity. Tetrahedron.

[3] Zimmer H, Rothe J (1959). Substituted γ-lactones. I. Preparation of α-substituted γ-butyrolactones by condensation of γ-butyrolactone with aldehydes. Hydrogenation of the condensation products. J Org Chem..

[4] Wang CH, Alluri S, Nikogosyan G, DeCarlo C, Monteiro C, Mabagos G, Feng HH, White AR, Bartolini M, Andrisano V, Zhang LK, Ganguly AK (2016). Novel synthesis of physovenine and physostigmine analogs. Tetrahedron Lett.

[5] Larson GL, de Perez RMB (1985). A two-step preparation of α-alkylidene γ-lactones from γ-lactones: a synthesis of (±)-ancepsenolide. J Org Chem.

[6] Richmond MH (1962). The effect of amino acid analogues on growth and protein synthesis in microorganisms. Microbiol Mol Biol Rev.

[7] Hartman MCT, Josephson K, Lin CW, Szostak JW (2007). An expanded set of amino acid analogs for the ribosomal translation of unnatural peptides. PLoS ONE.

[8] Auras R, Harte B, Selke S (2004). An overview of polylactides as packaging materials. Macromol Biosci.

[9] Chen W, Yang H, Wang R, Cheng R, Meng F, Wei W, Zhong Z (2010). Versatile synthesis of functional biodegradable polymers by combining ring-opening polymerization and postpolymerization modification via michael-type addition reaction. Macromolecules.

[10] Jiang X, Vogel EB, Smith MR, Baker GL (2008). “Clickable” polyglycolides: tunable synthons for thermoresponsive, degradable polymers. Macromolecules.

[11] Niaounakis M (2015). Biopolymers: applications and Trends.

[12] Freytag K, Säfken S, Wolter K, Namyslo JC, Hübner EG (2017). Hybrid single-chain nanoparticles via the metal induced crosslinking of N-donor functionalized polymer chains. Polym Chem..

[13] Zander NE, Orlicki JA, Rawlett AM (2010). Thermal and FTIR characterization of poly(4-vinylpyridine) crosslinked with metal salts.

[14] Yato M, Homma K, Ishida A (2001). Reduction of carboxylic esters to ethers with triethyl silane in the combined use of titanium tetrachloride and trimethylsilyl trifluoromethanesulfonate. Tetrahedron.

[15] Al-Shaal MG, Hausoul PJC, Palkovits R (2014). Efficient, solvent-free hydrogenation of α-angelica lactone catalysed by Ru/C at atmospheric pressure and room temperature. Chem Commun.

[16] Chanlaroff MBI (1884). Ueber das Butyrolacton und das α-Aethylbutyrolacton. Justus Liebigs Ann Chem..

[17] Farlow AJ, Bernhardt PV, De Voss JJ (2013). Synthesis of oxygenated cineole derivatives from cineole: utility of cytochrome P450_cin_ as an enantioselective catalyst. Tetrahedron..

[18] Wolfe S, Wilson MC, Cheng MH, Shustov GV, Akuche CI (2003). Cyclic hydroxamates, especially multiply substituted [1,2]oxazinan-3-ones. Can J Chem.

[19] Shimizu N, Tarui H, Mori N, Nishida R, Kuwahara Y (2003). *E*)-2-(2-Hydroxyethylidene)-6-methyl-5-heptenal (α-acariolal) and (*E*)-2-(2-hydroxyethyl)-6-methyl-2,5-heptadienal (β-acariolal), two new types of isomeric monoterpenes from C*aloglyphus polyphyllae* (acari: acaridae. Biosci Biotechnol Biochem.

[20] Nakamura Y, Fujimoto T, Ogawa Y, Namiki H, Suzuki S, Asano M, Sugita C, Mochizuki A, Miyazaki S, Tamaki K, Nagai Y, Inoue S, Nagayama T, Kato M, Chiba K, Takasuna K, Nishi T (2013). Lead optimization of 5-amino-6-(2,2-dimethyl-5-oxo-4-phenylpiperazin-1-yl)-4-hydroxyhexanamides to reduce a cardiac safety issue: Discovery of DS-8108b, an orally active renin inhibitor. Bioorg Med Chem.

[21] Takahasi Y, Hasegawa S, Izawa T, Kobayashi S, Ohno M (1986). Synthesis of cis-substituted β-lactams, potential intermediates for cis-carbapenems, from l-aspartic acid. Chem Pharm Bull.

[22] Boland S, Alen J, Bourin A, Castermans K, Boumans N, Panitti L, Vanormelingen J, Leysen D, Defert O (2014). Novel roflumilast analogs as soft PDE4 inhibitors. Bioorg Med Chem Lett.

[23] Huber W, Huber W (1967). Table I—acidic and basic strengths of various compounds in aqueous solution. Titrations in nonaqueous solvents.

[24] Ciolino LA, Mesmer MZ, Satzger RD, Machal AC, McCauley HA, Mohrhaus AS (2001). The chemical interconversion of GHB and GBL: forensic issues and implications. J Forensic Sci.

[25] Marques DS, Gil MH, Baptista CMSG (2013). Improving lactic acid melt polycondensation: the role of co-catalyst. J Appl Polym Sci..

