# Supplementary material for: Synthesis of γ-hydroxy-α-(arylmethyl)carboxylic acids from lactones: pathway to a structural motif derived from lactic acid and amino acid analogs?
Source: BMC Res Notes. 2019 Apr 2;12:197. doi: 10.1186/s13104-019-4232-1 (PMC6444536; doi:10.1186/s13104-019-4232-1)
Supplement: Supplementary file 1 — Additional file 1. Supporting information contains details concerning equipment, measurement parameters, suppliers of chemical substances, purification/drying of starting materials, procedure for polycondensation reactions, molecular weight of all polycondensation products, all analytical data for compounds 3, 4, 5 and 10, DOSY NMR spectra of 9 and the polycondensation product of lactic acid in presence of 3 after equimolar acidification as well as raw 1H and 13C NMR spectra of 3, 4, 5 and 10. [file 13104_2019_4232_MOESM1_ESM.pdf]

Additional file 1 for:

Synthesis of γ-hydroxy-α-(arylmethyl)carboxylic acids from lactones: pathway to a structural motif derived from lactic acid and amino acid analogs?

Nicole Panzier, Fabian Uhrner, Felix Lederle, Jan C. Namyslo and Eike G. Hübner*

Clausthal University of Technology

Institute of Organic Chemistry

Leibnizstr. 6

DE-38678 Clausthal-Zellerfeld

Germany

Phone: +49-5323-72-3834

Fax: +49-5323-72-2834

E-Mail: eike.huebner@tu-clausthal.de

Content

1. Experimental Section - Materials and Methods

2. Experimental Section - Combined Spectroscopic Data for **3**, **4**, **5** and **10**

3. Polycondensation

4. DOSY NMR Spectra

5. NMR Spectra

6. References

1. Experimental Section - Materials and Methods

All operations with air sensitive compounds were carried out at a vacuum line using Schlenk techniques. If not noted differently, chemicals were bought from *Sigma–Aldrich* and used as received. γ-Butyrolactone (*VWR International GmbH*) and benzaldehyde were distilled before use. Sodium methoxide was freshly prepared from methanol and sodium and dried in vacuo before use. α-Angelica lactone (*TCI Europe GmbH*), 1-methylpyrazol-4-carboxaldehyde (*TCI Europe GmbH*), titanium(IV) *n*-butoxide (*VWR International GmbH*) and Pd on activated carbon (Pd/C, 10% Pd) were used as received. D-,L-Lactic acid solution (80 wt-%, *Fluka Chemie GmbH*) was freeze-dried for 7 days at -60 °C and 0.03 mbar. Diethyl ether and tetrahydrofurane (THF) were passed through a PS-MD-4 solvent purification system (*inert technology*) for drying (<10 ppm H_2O). The concentration of NaOH and HCl solutions has been checked by titration before use. $CDCl_3$ (*Deutero GmbH*), D_2O (*Deutero GmbH*) and DMSO-d6 (*Deutero GmbH*) were used as received. A *Heraeus Labofuge 400R* (*ThermoFisher Scientific Messtechnik GmbH*) was used for centrifugation at 4500 rpm.

IR spectra: IR spectra were recorded with a *Bruker Alpha-T* FT-IR spectrometer (*Bruker Corporation*). For ATR measurements, a Platinum diamond-ATR unit was used.

NMR spectra: NMR spectra were recorded with a *Bruker Avance 400* (*Bruker Corporation*) (400 MHz (^{1}H), 100 MHz (^{13}C)) and *Avance III 600* (600 MHz (^{1}H), 150 MHz (^{13}C)) FT-NMR spectrometer. Chemical shifts are given in ppm relative to tetramethylsilane (δ = 0.0) or the residual solvent signal of the deuterated solvent.

DOSY NMR Spectra: Diffusion ordered (DOSY) NMR data were recorded on the *Bruker Avance III 600* spectrometer equipped with a broad band observed (BBO) probe with z-gradient. The spectrometer was additionally provided with the NMR thermometer hardware (*Bruker Corporation*) that ensured highly stable temperature conditions. Calibration of the gradient strength G was conducted by means of a deuterium oxide sample containing H_2O-traces and was found to be 0.548 $T\cdot m^{-1}$ for the BBO probe. The diffusion ordered spectra were performed in 3 mm NMR tubes Wilmad 335 (*Wilmad-LabGlass*) under calibrated (methanol thermometer) and carefully stabilized temperature conditions, i.e. T = 298±0.1 K. The probe head nitrogen gas flow was adjusted to 800 $L\cdot h^{-1}$. Optimized pulse repetition

delays (d1 = 10 s) were obtained from inversion-recovery experiments. Sample spinning was used in order to avoid convection. The applied pulse sequence was the DOSY Oneshot experiment creating a series of 16 1-D spectra with squared increase of the gradient field strength from 10 to 80 % [1]. The intergradient delay (diffusion time) Δ (d20) was set to 0.10 s. The corresponding length of the gradient pulse (p30; $\delta/2$) was adjusted to 0.9 ms (**1**) or 1.25 ms (polycondensation products), respectively. Each single spectrum was processed with a line-broadening factor (LB) of 1.0 Hz. The DOSY data were analyzed with the DOSY Toolbox in order to calculate diffusion coefficients and generate the common DOSY plot [2].

Mass spectra: Electrospray ionization (ESI) mass spectra were recorded with an *HP 1100 Series* (*Hewlett-Packard/Agilent Technologies, Inc.*) LC/MS mass spectrometer. Negative ion mode was applied.

High-resolution electrospray ionization mass spectra (HR-MS (ESI)) were recorded on an *IMPACT II* (*Bruker Corporation)* mass spectrometer with negative ion mode.

Electron impact (EI) mass spectra were recorded with a *Varian 320 MS TQ* (*Agilent Technologies, Inc.*) mass spectrometer at 20 eV.

SEC measurements: Size exclusion chromatography was performed with a setup equipped with a *Waters 515* HPLC pump (*Waters GmbH*), *Knauer Smartline* RI detector *2300* (*Knauer Wissenschaftliche Geräte GmbH*) and 4 × 20 µm mixed-A columns from *Polymer Laboratories* (*Agilent Technologies, Inc.*). Tetrahydrofuran (THF) with a flow rate of 1 $mL \cdot min^{-1}$ at 25 °C was used as eluent. Molecular weights were obtained relative to polystyrene (PS) calibration.

2. Experimental Section - Combined Spectroscopic Data for 3, 4, 5 and 10

2.1 Sodium 4-hydroxy-2-((*N*-methylpyrazol-4-yl)methyl)pentanoate (3)

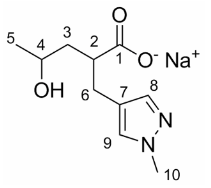

^{1}H NMR (400 MHz, DMSO-d6): δ =7.35 (s, 1H, H-9), 7.16 (s, 1H, H-8), 6.73 (bs, 1H, OH), 3.72 (s, 3H, H-10), 3.59 (qdd, ^{3}J = 9.1 Hz, ^{3}J = 6.0 Hz, ^{3}J = 3.2 Hz, 1H, H-4), 2.67 (dd, ^{2}J = 14.3 Hz, ^{3}J = 5.8 Hz, 1H, H-6), 2.38 (dd, ^{2}J = 14.3 Hz, ^{3}J = 7.3 Hz, 1H, H-6'), 2.17 (dddd, ^{3}J = 9.7 Hz, ^{3}J = 7.3 Hz, ^{3}J = 5.8 Hz, ^{3}J = 3.2 Hz, 1H, H-2), 1.45 (ddd, ^{2}J = 13.6 Hz, ^{3}J = 9.7 Hz, ^{3}J = 9.1 Hz, 1H, H-3), 1.27 (ddd, ^{2}J = 13.6 Hz, ^{3}J = 3.2 Hz, ^{3}J = 3.2 Hz, 1H, H-3'), 0.95 (d, ^{3}J = 6.0 Hz, 3H, H-5) ppm. ^{13}C NMR (100 MHz, DMSO-d6): δ = 180.1 (1C, C-1), 138.5 (1C, C-8), 129.1 (1C, C-9), 120.2 (1C, C-7), 66.9 (1C, C-4), 47.7 (1C, C-2), 41.5 (1C, C-3), 38.2 (1C, C-10), 28.5 (1C, C-6), 24.7 (1C, C-5) ppm. IR (ATR): 3267, 2962, 2930, 1559 (asym. CO_2^-), 1397 (sym. CO_2^-), 1309, 1129, 1058 cm^{-1}. ESI-MS: m/z = 211.1 (100 %) [M–Na]$^-$, 445.2 (30 %) [2M–Na]$^-$, 679.2 (10 %) [3M–Na]$^-$.

2.2 Sodium 4-hydroxy-2-(phenylmethyl)butanoate (4)

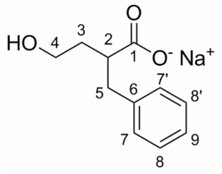

^{1}H NMR (400 MHz, D_2O): δ =7.36 (m, 2H, H-8/8'), 7.26 (m, 2H, H-7/7'), 7.25 (m, 1H, H-9), 3.64 – 3.52 (m, 2H, H-4), 2.84 (dd, ^{2}J = 13.5 Hz, ^{3}J = 9.0 Hz, 1H, H-5), 2.76 (dd, ^{2}J = 13.5 Hz, ^{3}J = 6.2 Hz, 1H, H-5'), 2.63 – 2.55 (m, 1H, H-2), 1.85 – 1.76 (m, 1H, H-3), 1.74 – 1.65 (m, 1H, H-3') ppm. ^{13}C NMR (100 MHz, D_2O): δ = 182.9 (1C, C-1), 139.5 (1C, C-6), 127.9 (2C, C-7/7'), 127.4 (2C, C-8/8'), 125.1 (1C, C-9), 59.1 (1C, C-4), 46.7 (1C, C-2), 37.7 (1C, C-5), 33.6 (1C, C-3) ppm. IR (ATR): 2914, 2876, 2854, 1752, 1586, 1554 (asym. CO_2^-), 1494, 1438, 1407 (sym. CO_2^-), 1367, 1334, 1290, 1267, 1199, 1163, 1077, 1055, 1036, 933, 912, 865, 841, 823, 769, 744, 695, 647, 570, 51, 617, 466, 414 cm^{-1}. HR-MS (ESI): [M–Na]$^-$, found 193.0872. $C_{11}H_{13}O_3$ requires 193.0870.

2.3 Sodium 4-hydroxybutanoate (5)

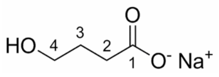

^{1}H NMR (400 MHz, D_2O): δ =3.58 (t, ^{3}J = 6.7 Hz, 2H, H-4), 2.23 (t, ^{3}J = 7.3 Hz, 2H, H-2), 1.78 (tt, ^{3}J = 7.3Hz, 6.7 Hz, 2H, H-3) ppm. ^{13}C NMR (100 MHz, D_20): δ = 182.8 (1C, C-1), 61.5 (1C, C-4), 33.8 (1C, C-2), 28.4 (1C, C-3) ppm. IR (ATR): 3310, 2959, 2941, 2876, 1554 (asym. CO_2^-), 1476, 1449, 1405 (sym. CO_2^-), 1328, 1272, 1229, 1157, 1064, 1014, 945, 869, 920, 772, 749, 635, 575, 548, 482 cm^{-1}. HR-MS (ESI): [M–Na]$^-$, found 103.0402. $C_4H_7O_3$ requires 103.0401.

2.4 Sodium (*E*)-2-((*N*-methylpyrazol-4-yl)methylene)-4-oxopentanoate (10)

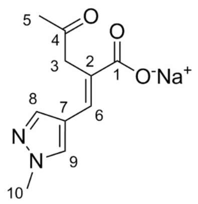

^{1}H NMR (400 MHz, DMSO-d6): δ = 7.77 (s, 1H, H-9), 7.49 (s, 1H, H-8), 7.30 (s, 1H, H-6), 3.81 (s, 3H, H-10), 3.46 (s, 2H, H-3), 2.04 (s, 3H, H-5) ppm.

^{13}C NMR (100 MHz, DMSO-d6): δ = 207.3 (1C, C-4), 171.4 (1C, C-1), 139.0 (1C, C-8), 132.3 (1C, C-2), 130.1 (1C, C-9), 123.9 (1C, C-6), 118.4 (1C, C-7), 44.8 (1C, C-3), 38.5 (1C, C-10), 29.1 (1C, C-5) ppm. IR (ATR): 3123, 2989, 2942, 2559, 1739, 1697, 1652 (C=O), 1546 (asym. CO_2^-), 1414, 1358, 1304, 1206, 1159, 1039, 1004 cm^{-1}. EI-MS: m/z = 253.1 (10 %) [M+Na]$^+$, 189.9 (100 %) [M–ONa]$^+$, 120.1 (100 %) [M–CO_2Na–CH_3CO]$^+$

3. Polycondensation

3.1 General procedure for polycondensation of lactic acid adapted from ref. [3]

5.0 g (taken as 0.056 mol) pre-dried (freeze-drying) lactic acid and if appropriate 5 – 10 mol-% of the comonomer in form of the equimolarly acidified mixture of sodium 4-hydroxy-2-((*N*-methylpyrazol-4-yl)methyl)pentanoate (**3**) are weighed in a 50 mL Schlenk tube under a nitrogen atmosphere. The mixture is heated to 170 °C (or 150 °C, respectively) and stirred for 16 h. Subsequently, 1 mol-% (vs. sum of monomers) of titanium(IV) *n*-butoxide is added via a syringe. The temperature is raised to 180 °C (or 155 °C, respectively) and stirring continued for 1h. Subsequently, the reaction vessel is slowly evacuated to 30 mbar during 7 h. The pressure is reduced further to roughly 1 mbar and heating/stirring continued at these conditions for 16 h. Water and evaporating lactic acid are removed by a cold trap. After the reaction time has completed, the Schlenk tube is cooled down and the crude reaction product is isolated.

For further purification, the crude mixture is dissolved in a little chloroform and precipitated in a fourfold excess of ethanol. The precipitate is isolated by centrifugation and the resulting slightly beige powder dried in vacuo. In case of polycondensation reactions in presence of **3,** the supernatant solution of the centrifugation step is dried in vacuo to isolate an additional fraction.

3.2 Molecular weight of polycondensation products

Table S1 Summary of polycondensation reactions

Entry	*Fraction of **3***[a]	*T*	*t*	M_n[b]	M_w/M_n
1	-	155 °C	40 h	6 600	1.4
2	-	180 °C	40 h	10 600	1.5
3	5 mol-%	180 °C	40 h	2 600[c]	-[d]
4	10 mol-%	180 °C	168 h	4 100[c]	-[d]

[a] After addition of equimolar amounts of HCl. [b] From SEC rel. PS calibration. [c] Only the highest molecular weight signal has been analyzed. [d] Due to the formation of several oligomers, M_w/M_n is not calculated.

3.3 Molecular weight distribution

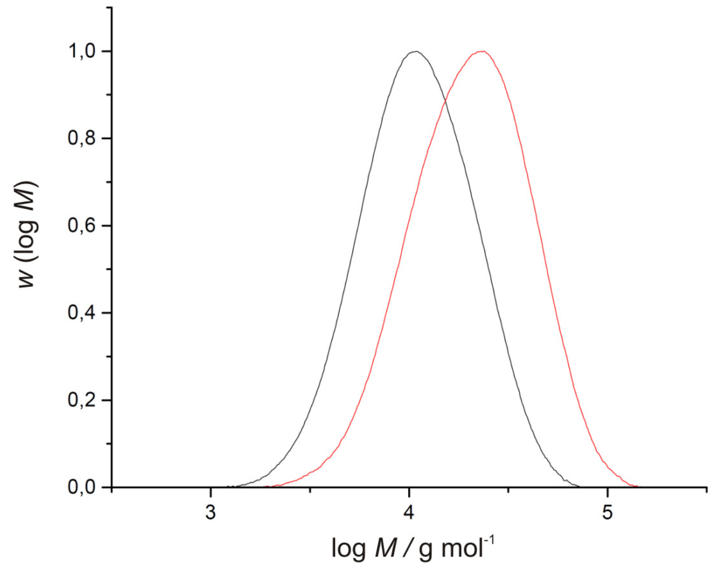

Fig. S1 Molecular weight distribution of polylactic acid**.** Obtained by polycondensation of pure lactic acid according to procedure 3.1 at 180 °C (red, entry 2 in Additional file 1: Table S1) and 155 °C (black, entry 1 in Additional file 1: Table S1) rel. to PS standard.

4. DOSY NMR spectra

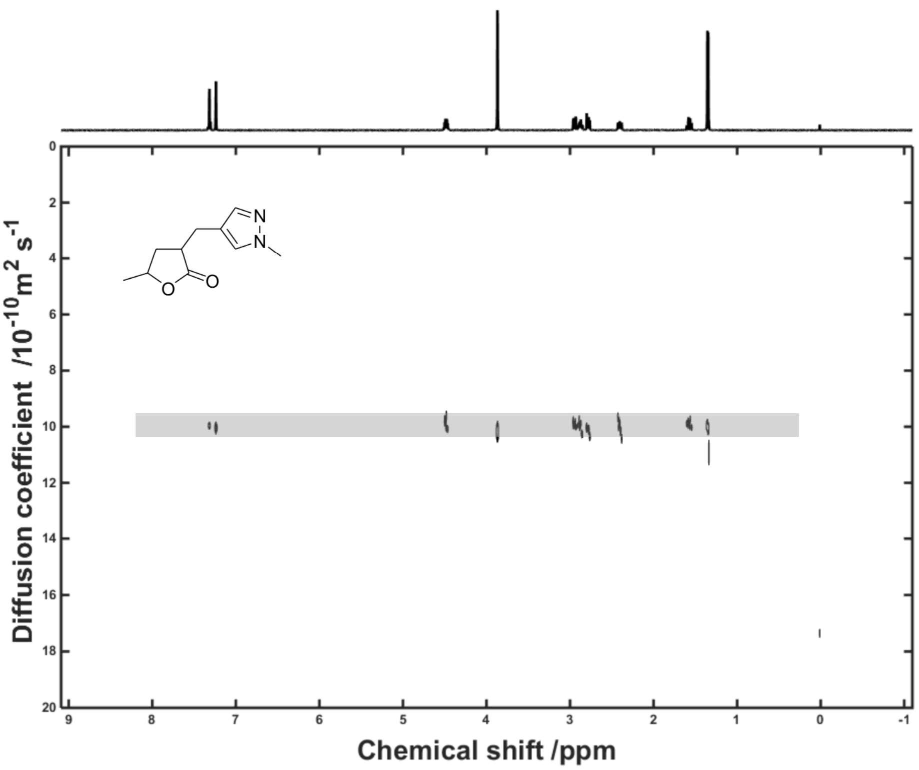

Fig. S2 Diffusion ordered (DOSY) NMR plot of 5-methyl-3-((*N*-methylpyrazol-4-yl)methyl)dihydrofuran-2-one (**9**). Measured in $CDCl_3$.

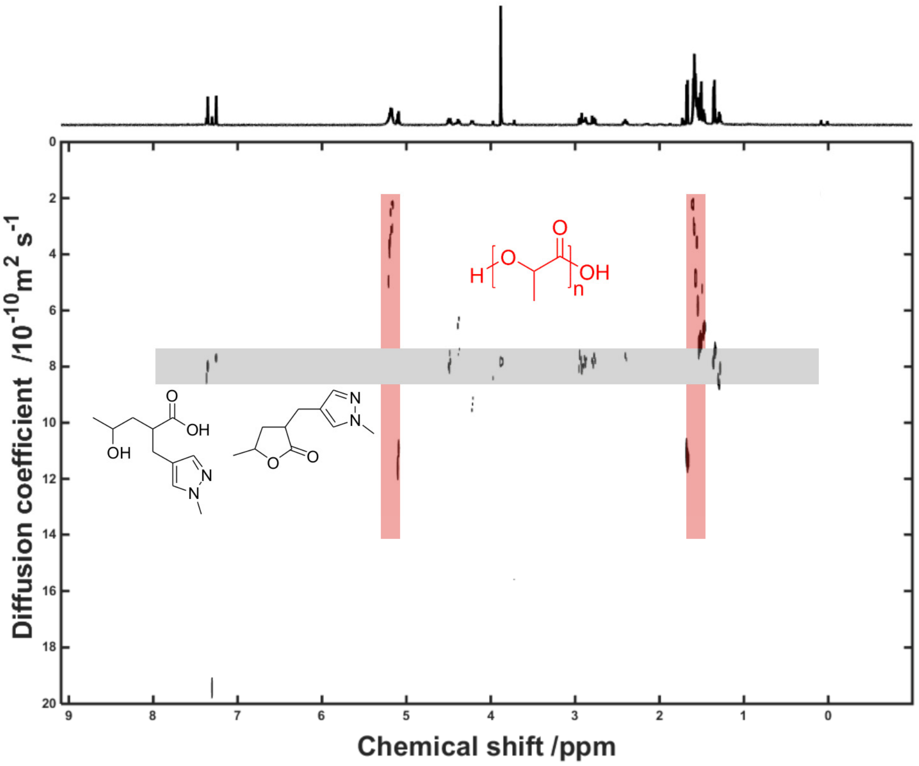

Fig. S3 Diffusion ordered (DOSY) NMR plot of the crude reaction product of the polycondensation of lactic acid in presence of 5 mol-% of the equimolarly acidified mixture of **3** (entry 3 in Additional file 1: Table S1). Several oligomers of lactic acid (red) and only low molecular weight compounds related to **3** (grey) are identified. Measured in $CDCl_3$.

5. NMR spectra

^{1}H NMR spectrum of sodium (*E*)-2-((*N*-methylpyrazol-4-yl)methylene)-4-oxopentanoate (**10**) in DMSO-d6:

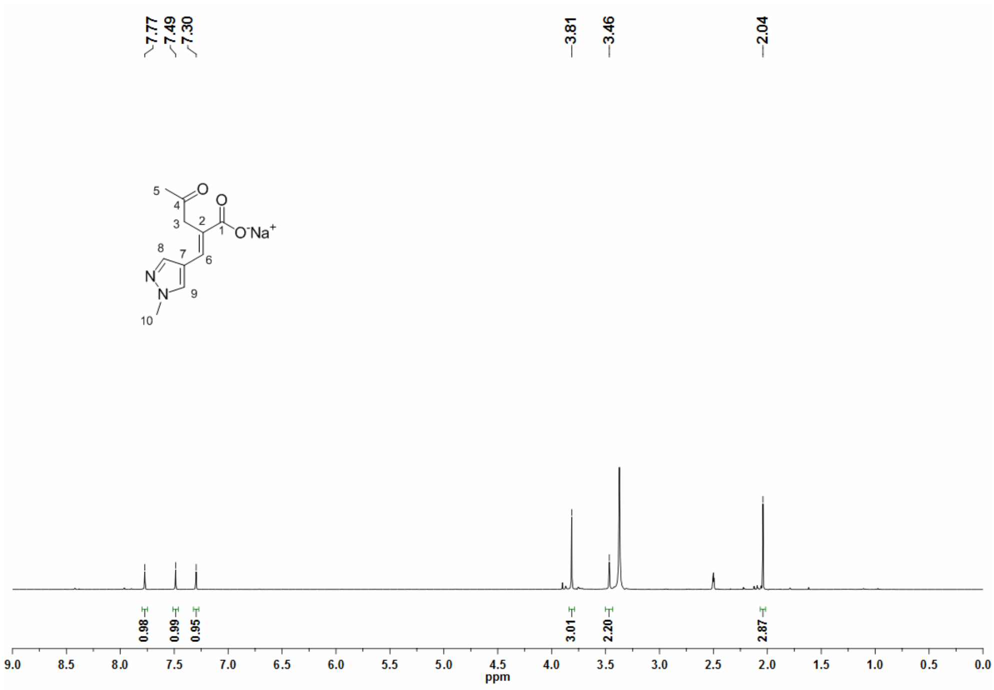

^{13}C NMR spectrum of sodium (*E*)-2-((*N*-methylpyrazol-4-yl)methylene)-4-oxopentanoate (**10**) in DMSO-d6:

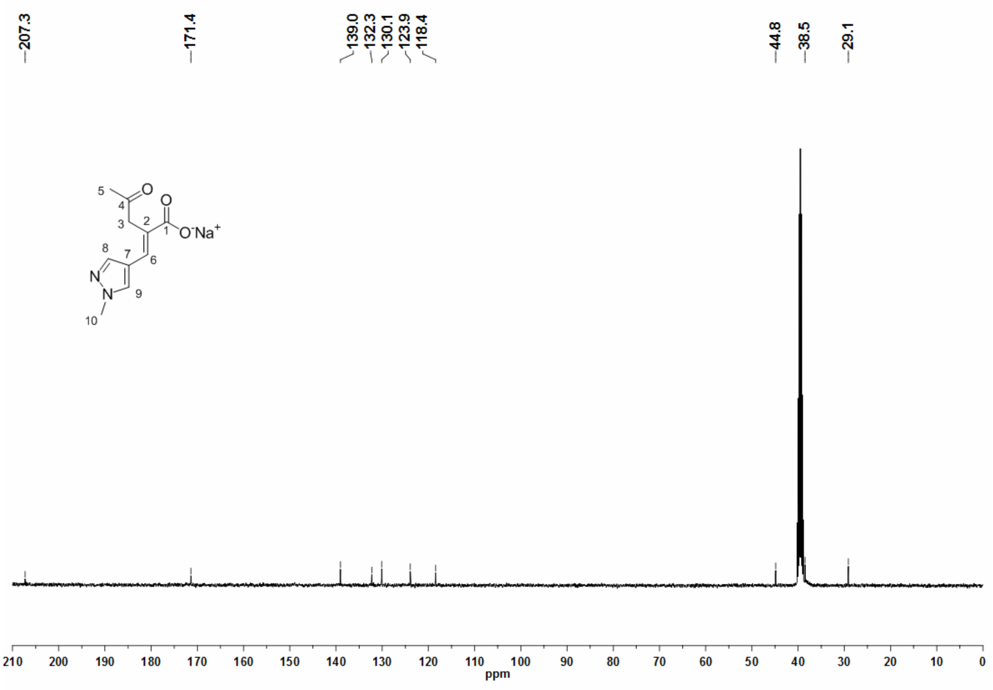

^{1}H NMR spectrum of sodium 4-hydroxy-2-((*N*-methylpyrazol-4-yl)methyl)pentanoate (**3**) in DMSO-d6:

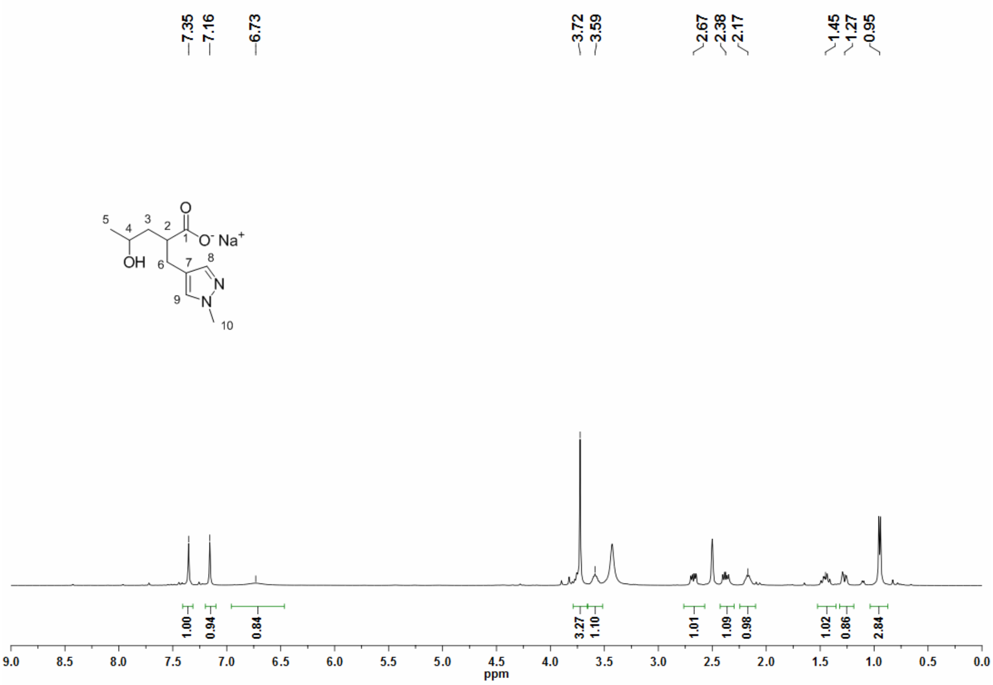

^{13}C NMR spectrum of sodium 4-hydroxy-2-((*N*-methylpyrazol-4-yl)methyl)pentanoate (**3**) in DMSO-d6:

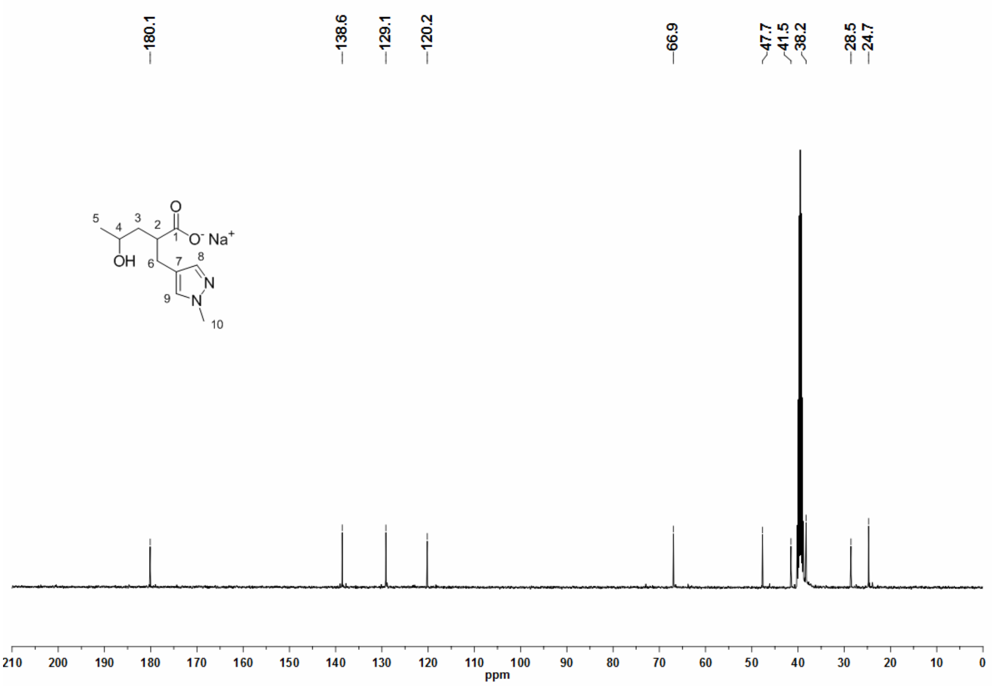

^{1}H NMR spectrum of sodium 4-hydroxy-2-(phenylmethyl)butanoate (**4**) in D_2O:

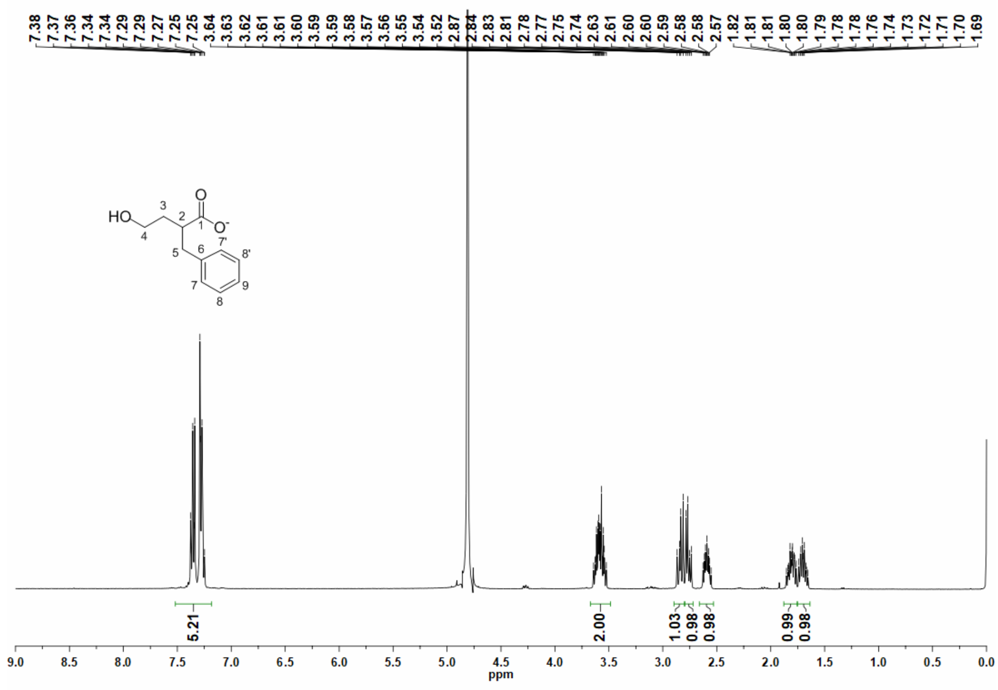

^{13}C NMR spectrum of sodium 4-hydroxy-2-(phenylmethyl)butanoate (**4**) in D_2O:

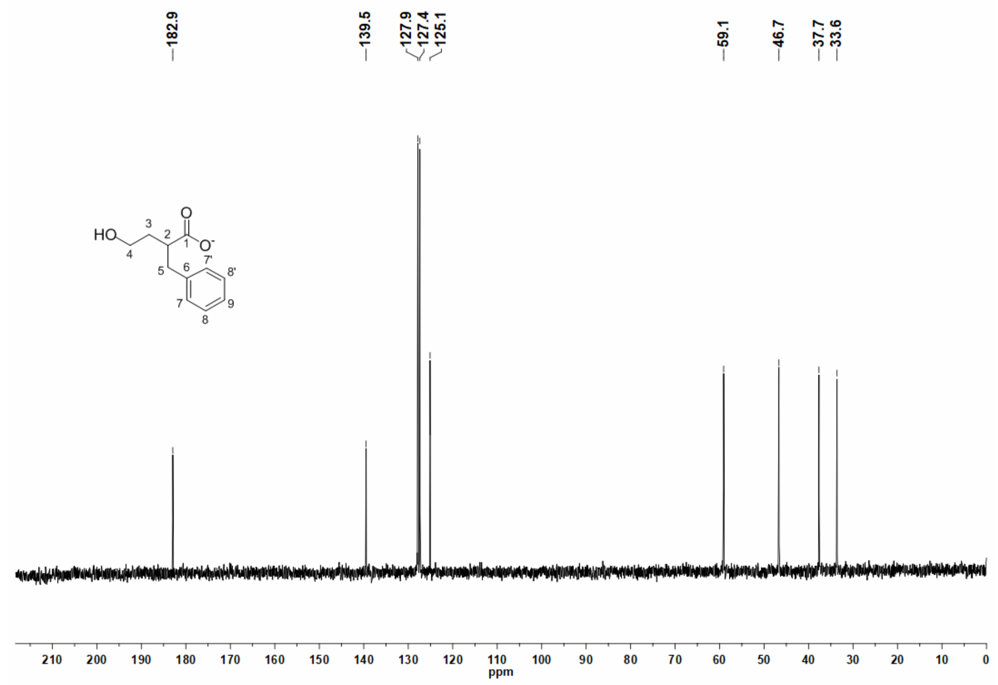

^{1}H NMR spectrum of sodium 4-hydroxybutanoate (**5**) in D_2O:

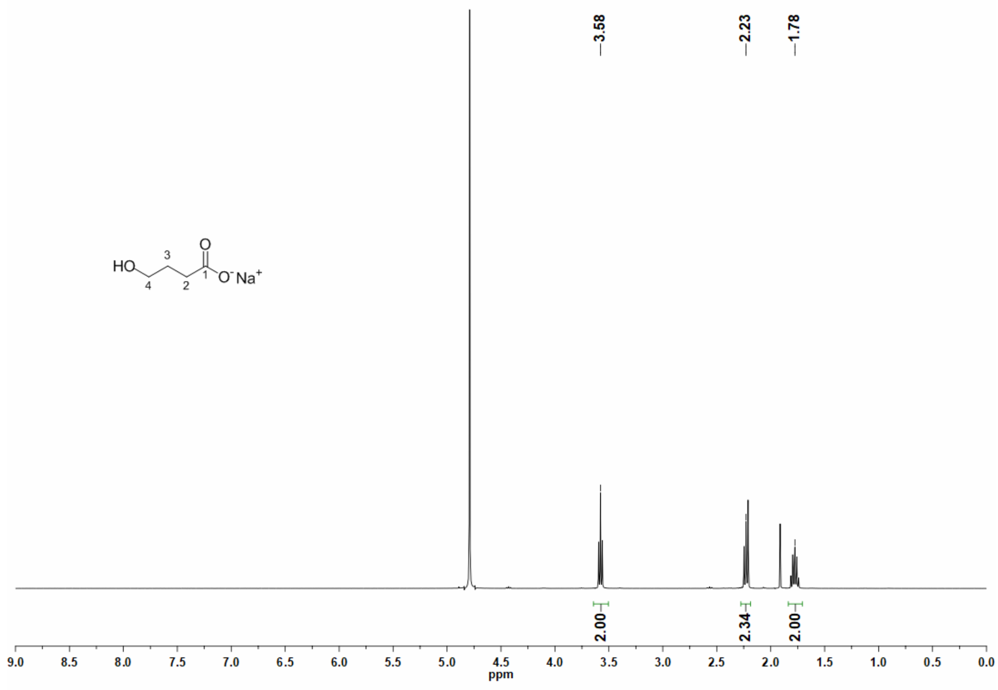

^{13}C NMR spectrum of sodium 4-hydroxybutanoate (**5**) in D_2O:

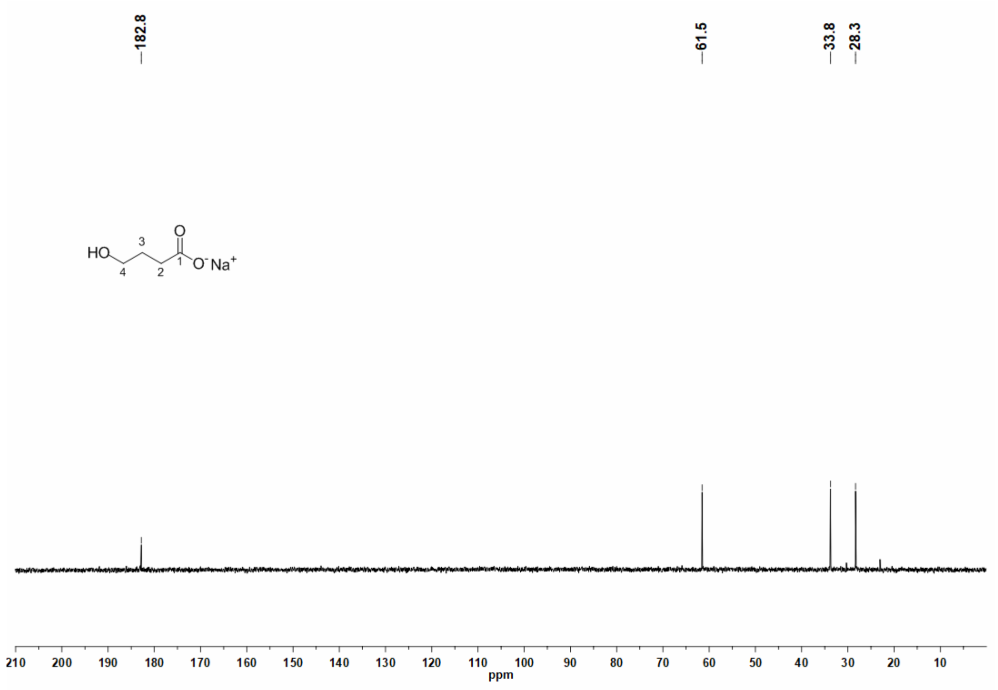

6. References

[1] Pelta MD, Morris GA, Stchedroff MJ, Hammond SJ. A one-shot sequence for high-resolution diffusion-ordered spectroscopy. Magn Reson Chem. 2002;40:S147–52. https://doi.org/10.1002/mrc.1107.

[2] Nilsson M. The DOSY Toolbox: A new tool for processing PFG NMR diffusion data. J Magn Res. 2009;200:296-302. https://doi.org/10.1016/j.jmr.2009.07.022.

[3] Marques DS, Gil MH, Baptista CMSG. Improving lactic acid melt polycondensation: The role of co-catalyst. J Appl Polym Sci. 2013;128:2145–51. https://doi.org/ 10.1002/APP.38413.
